# Nanoparticle-Mediated Pulmonary Drug Delivery: A Review

**DOI:** 10.3390/ijms15045852

**Published:** 2014-04-08

**Authors:** Mukta Paranjpe, Christel C. Müller-Goymann

**Affiliations:** Institute for Pharmaceutical Technology, TU Braunschweig, Mendelssohnstr. 1, 38106 Braunschweig, Germany; E-Mail: mukta.paranjpe@tu-bs.de

**Keywords:** nanoparticles, SLN, toxicity, lung cell models, aerosol, nebulization, lung disease

## Abstract

Colloidal drug delivery systems have been extensively investigated as drug carriers for the application of different drugs via different routes of administration. Systems, such as solid lipid nanoparticles, polymeric nanoparticles and liposomes, have been investigated for a long time for the treatment of various lung diseases. The pulmonary route, owing to a noninvasive method of drug administration, for both local and systemic delivery of an active pharmaceutical ingredient (API) forms an ideal environment for APIs acting on pulmonary diseases and disorders. Additionally, this route offers many advantages, such as a high surface area with rapid absorption due to high vascularization and circumvention of the first pass effect. Aerosolization or inhalation of colloidal systems is currently being extensively studied and has huge potential for targeted drug delivery in the treatment of various diseases. Furthermore, the surfactant-associated proteins present at the interface enhance the effect of these formulations by decreasing the surface tension and allowing the maximum effect. The most challenging part of developing a colloidal system for nebulization is to maintain the critical physicochemical parameters for successful inhalation. The following review focuses on the current status of different colloidal systems available for the treatment of various lung disorders along with their characterization. Additionally, different *in vitro*, *ex vivo* and *in vivo* cell models developed for the testing of these systems with studies involving cell culture analysis are also discussed.

## 1. Introduction

**Figure 1.**
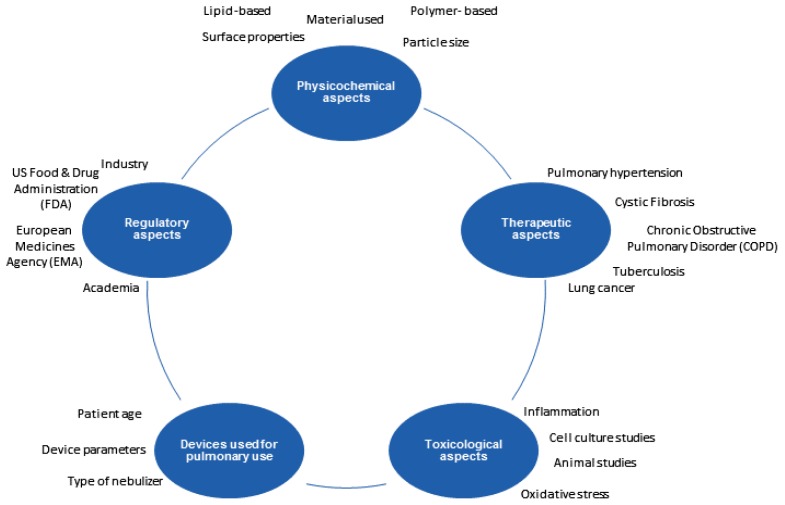
Complex interplay of parameters in the research and development of pulmonary drug delivery systems.

Lungs are an attractive target for the pulmonary administration of active pharmaceutical ingredients (APIs) in the form of various drug delivery systems [1–3]. Additionally, this route offers many advantages over conventional per oral administration, such as a high surface area with rapid absorption due to high vascularization and circumvention of the first pass effect [2]. This selectivity allows targeted drug delivery and, hence, reduces the side effects [4,5]. Colloidal drug delivery systems have extensively been investigated as drug carrier systems for the application of different drugs via different routes of administration. Solid lipid nanoparticles (SLN) are one of the most interesting colloidal systems that has studied for more than a decade [6,7]. SLN are aqueous nanoscale suspensions prepared mainly from phospholipids and the triglycerides of physiological tolerability [8–10]. Along with SLN, biodegradable polymeric nanoparticles are also attaining importance due to the sustained release of APIs. These systems are an ideal platform for lipophilic drugs, which have poor solubility in aqueous systems. Thus, increased solubility in a lipophilic matrix adds a positive effect on the pharmacokinetics and therapeutic efficacy. Nanoparticle-mediated drug delivery systems open new perspectives by modifying the physical properties of the particles, such as increasing the drug solubility, encapsulation efficacy and surface alterations to enhance the drug release profiles and to obtain a maximum effect [11–13]. Although most of these nanosystems have therapeutic effects, toxicological effects have to be considered, as well. The toxicological testing in different cell culture models, *i.e.*, *in vitro*, *ex vivo* and *in vivo* models, is necessary to determine a safe dose. Even though the results from the cell culture models cannot be directly extrapolated to an *in vivo* situation of an individual patient, the testing of nanosystems in such models is essential to reduce the risk of adverse reactions or toxic effects. The choice of the inhalation device in a specific patient population also plays a vital role in nanoparticle-mediated drug delivery systems for pulmonary application. The complex relationship between nanoparticle systems and various parameters to be considered during formulation development is illustrated in Figure 1.

## 2. Anatomy and Physiology of the Lungs

### 2.1. Anatomy of the Lungs

Lungs are responsible for the gas exchange and supply of oxygen to all the cells. The lungs consist of a total of five lobes, the right lung consisting of three and the left lung of two lobes. The interior of the lungs is comprised of bronchi and smaller air passages, alveoli, blood vessels and lymph tissue. The bronchi are further divided into primary and secondary bronchi and bronchioles and, finally, the alveoli. Lungs have over 300 million alveoli. Furthermore, each alveolus is lined with pulmonary capillaries, thus forming a vast network comprising over 280 billion capillaries, giving rise to a vast surface area of almost 70 m^2^ available as the blood-gas barrier. The alveolar gas exchange majorly occurs at the interface consisting of alveolar epithelium, endothelium and interstitial cell layers. The alveolar wall is made up of two types of alveolar epithelial cells, namely (pneumonocytes) Type I and Type II. Between the capillaries and the alveolar epithelium, there exists a single endothelial layer. The distance between the alveoli and capillaries is so small, about 0.5 μm, that owing to this extreme thinness of the blood-gas interface, gas exchange is facilitated by diffusion at the interface. The alveoli are coated with a layer of alveolar fluids and mucus, which is majorly composed of phospholipids and surface proteins. This phospholipid surfactant layer at the alveoli reduces the surface tension and is essential for the proper functioning of the gas exchange. These distal respiratory passages are supported by a thin layer of connective tissue. This layer is surrounded with different cells, like macrophages, fibroblasts, nerves, as well as lymph vessels. This serves as an ideal location for the administration of drugs with access to the pulmonary, as well as the lymphatic system [14,15].

### 2.2. Deposition of the Particles

The deposition of particles in the different regions of the lungs depends on the particle size of the formulation. Based on the particle size, three different mechanisms of drug deposition are defined, namely impaction, sedimentation and diffusion [16]. In impaction, the aerosol particles pass through the oropharynx and upper respiratory passages at a high velocity. Due to the centrifugal force, the particles collide with the respiratory wall and are deposited in the oropharynx regions. This mechanism is generally observed for dry powder inhalation (DPI) and metered dose inhalators (MDI), with particles sizes greater than 5 μm. In case of the DPI, the inspiratory effort of the patient plays an important role in the deposition. If the force of inhalation is insufficient, the dry powder deposits in the upper airways, owing to the mass of the particles and the inertial forces. For the MDI and despite the high speed of the generated aerosol, high particle sizes also tend to lead to the deposition of the particles mostly in the upper respiratory tract region. Gravitational forces are predominantly responsible for the sedimentation of particles. Particles with sufficient mass and sizes between one to 5 μm are deposited in the smaller airways and bronchioles, where they are deposited slowly, provided a sufficiently long time span. Therefore, sedimentation is also influenced by the breathing pattern. Slow breathing provides a sufficient time span for sedimentation. Apart from impaction and sedimentation, Brownian motion plays a major role in the deeper alveolar areas of the lungs. The Brownian motion of the surrounding molecules of the aqueous lung surfactant causes a random movement of the particles. Upon contact with the lung surfactant, the dissolution of API in alveolar fluid is essential for diffusion. Additionally, the concentration gradient also influences the diffusion process. Particles smaller than one to 0.5 μm are deposited in the alveolar region, while most of the particles, owing to smaller sizes, are exhaled [17]. For nanoparticulate systems, sedimentation is the most attractive method of particle deposition. Nanoparticulate systems, after being released from an aerosol, form aggregates in the micrometer size range. These aggregates are believed to have a sufficient mass to sediment and stay in the bronchiolar region for a longer time, hence achieving the desired effect. Apart from the mechanisms, parameters, such as the particle size of the aerosol, particle morphology and geometry, along with surface properties, play an important role in deposition phenomena. Furthermore, breathing frequency and the holding of breaths, humidity, air velocity and tidal volume also are vital factors influencing the deposition [17]. The correlation of particle size and the area of drug deposition is given in Table 1.

### 2.3. Clearance of the Particles

The upper airways (from the trachea till the tertiary bronchi) are lined with a thick mucus film, which acts as a protective layer in order to trap and clear the particles. The mucociliary movements clear the foreign particles immediately before they can move to lower areas of the lung by either coughing or swallowing [18,19]. The clearance in this region also depends on the number of cilia and the ciliary beat frequency, as well as the quality and quantity of mucus [16]. In the deeper areas of the lungs, *i.e.*, the alveolar region, the transport mechanism is believed to be more complex. The alveolar lining consists of a variety of proteins and lipids, which act as a barrier for the transport of the molecules. Along with the alveolar lining, the tight junctions present at the epithelial cell serve as the primary barrier for the transport to occur. The transporter proteins play a vital role in the transport of the API via active absorption or passive diffusion, depending on the nature and chemical structure of the API. Another important aspect in this region is the clearance of molecules by the alveolar macrophages, which needs to be taken into consideration in the drug transport mechanisms [16,19]. The molecules that are able to cross the barrier are either taken up by the cells and further absorbed into the systemic circulation or phagocytized by the alveolar macrophages.

**Figure 2.**
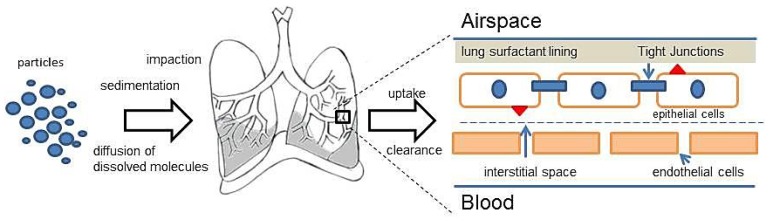
The deposition mechanism and uptake of particles in the lungs along with different cell types.

Hence, for understanding the uptake and clearance mechanisms of drug formulation, it is essential to understand the physiology of the lungs. In spite of advances in the formulation development, there is still some lack of information with respect to the exact uptake, transport and clearance of particles in the alveolar epithelium and how the API molecules reach the systemic circulation. Although several *in vitro* models have been established for studying the uptake and permeation of the APIs in the pulmonary epithelium (air-liquid interface models), there are still open questions with respect to the behavior of the cells in a diseased condition. The interplay of different cell types involved along with the deposition mechanisms is illustrated in Figure 2.

The lungs, being in contact with the air, are susceptible to numerous diseases and disorders, ranging from respiratory infections to genetic and lifestyle diseases. The most common diseases include asthma, pulmonary hypertension, chronic obstructive pulmonary disorder (COPD), acute respiratory distress syndrome (ARDS) in infants, cystic fibrosis, lung infections, like pneumonia, tuberculosis and chronic lung cancers.

## 3. Nanoparticle-Based Systems for Pulmonary Application

### 3.1. Solid Lipid Nanoparticles (SLN) and Solid Lipid Microparticles (SLM)

Solid lipid nanoparticles (SLN) have extensively been studied for a long time for potential pulmonary drug delivery. SLN are nanoscale aqueous suspensions prepared from physiological lipids, primarily triglycerides and phospholipids. As the formulations are based on using physiological components, they are less toxic and, as a result, more acceptable for pulmonary drug delivery. Phospholipids are present ubiquitously in the deep areas of lung and are essential for the functioning of the breathing mechanism. Phospholipid-based surfactant proteins present at the alveolar surface are essential for maintaining optimal surface tension and reducing friction in the lung tissue [20]. Nassimi *et al*. [8,9] demonstrated the use of phospholipid-and triglyceride-based SLN in the ratio of 30:70 for potential pulmonary applications. They also evaluated the toxicity profile of these SLN in *in vitro*, *ex vivo* and *in vivo* models along with cytokine activation measurements. They observed no activation of pro-inflammatory cytokines (TNF-α and chemokine-KC) when the SLN were administered in mice via nebulization [8,9]. Paranjpe *et al*. observed the toxicological effect of sildenafil-loaded SLN in *in vitro* and *ex vivo* models using lung and heart tissues from murine models for pulmonary administration for the treatment of pulmonary arterial hypertension [10]. In these studies, high median lethal dose 50% *i.e.*, LD_50_ values were observed in heart tissue models in comparison with the lung tissue model [10]. Owing to the similar lipid matrix base utilized in the studies by Nassimi and Paranjpe, these SLN can be suitable for the pulmonary administration of API for the treatment of pulmonary diseases [9,10]. In another study performed recently, quercetin-loaded solid lipid microparticles (SLM) were characterized for physicochemical analysis and studied for potential treatment in asthma for the anti-oxidant and anti-inflammatory properties of the flavonoid, quercetin [21]. This quercetin-SLM was manufactured using glyceryl trimyristate and soy lecithin and displayed acceptable mean mass aerodynamic diameter (MMAD) values. From the *in vitro* deposition studies, the authors observed that particles were stable after nebulization and were predominantly deposited in deep areas of the lung. Furthermore, in another study by Wang *et al*. [22], they manufactured stearic acid- and lecithin-based SLN loaded with curcumin. Curcumin-loaded SLN were produced using the solvent injection method for the possible treatment of asthma. These SLN were found to be stable between sizes ranging between 190–200 nm. From an *in vivo* study involving ovalbumin-induced asthma, they observed that the cytokine levels decreased in the curcumin-treated group *vs.* the untreated group [22]. Additionally, the suppression of the airway hyper-response and inflammatory cell infiltration, along with the reduced expression of the cytokines, such as interleukin-4 and interleukin-13, due to curcumin-SLN, suggests the suitability of curcumin-SLN for the treatment of asthma [22].

For the treatment of lung infections, various drugs have been investigated. SLN with amikacin, an aminoglycoside antibiotic, was manufactured using cholesterol as the lipid by a high pressure homogenization method [23]. The authors also studied the biodistribution of amikacin SLN after pulmonary administration. For the biodistribution assessment, radioisotope technetium (^99m^Tc) labeled amikacin was used to track the deposition of amikacin in different tissues. For *in vivo* studies, rats were treated with and without radiolabeled amikacin via the pulmonary route and intravenous route. From gamma scintigraphy analysis, it was observed that ^99m^Tc-amikacin SLN were found to last longer in the lungs via pulmonary administration as compared to the intravenous route. Additionally, it was observed that the deposition of ^99m^Tc-amikacin SLN was higher in lungs as compared to kidneys [23]. From these experiments, it is clear that radiolabeling assessments using gamma scintigraphy can provide vast information with respect to the deposition of an API in different tissues. It would be certainly useful for using diseased conditions in animal subjects, which can lead to more realistic information in an *in vivo* model. Apart from SLN, certain fatty acids can improve the drug solubility and increase biodistribution. In studies performed by Mussi *et al*. [24], doxorubicin SLN was investigated for anti-cancer activity. In order to enhance the drug encapsulation in the lipid melt, a polyunsaturated fatty acid, docosahexaenoic acid (DHA), was used. The drug encapsulation efficacy was observed to improve from 36% to 99%. Apart from DHA, triethanolamine was used to increase the solubility of doxorubicin in the lipid melt. Solubility enhancers can certainly improve the formulation efficacy, achieving maximum uptake and, hence, maximizing the cytotoxicity of doxorubicin [24].

Considering the advantages of SLN, like the low toxicity and use of physiological phospholipid components, they still have remained as a popular drug delivery system, even after more than two decades. Drugs that have been incorporated with the SLN and SLM for pulmonary administration are listed in Table 2.

### 3.2. Polymeric Nanoparticles

Polymers are gaining rapid importance for pulmonary drug delivery. Several polymers have been investigated for pulmonary application. Polymers have numerous advantages, like modified surface properties, high encapsulation of the drug and protection of the drug from degradation, prolonged drug delivery and a long shelf life. For therapeutic purposes, the most commonly used polymers include poly(lactic acid) (PLA), poly(lactic-*co*-glycolic acid) (PLGA), poly(ɛ-caprolactone) (PCL), alginate, chitosan and gelatin base [11]. These are modified in their chemical and surface properties in order to make them biodegradable [71,75]. Recently, from a study performed by Beck-Broichsitter and colleagues, the influence of polymeric nanoparticles on a pulmonary surfactant and its surface properties was assessed. The authors also compared the effect of synthetic and biodegradable polymeric nanoparticles on the pulmonary surfactant. They found dose-dependent changes in the surface tension of the pulmonary surfactant. Several studies have been performed using chitosan-based nanoparticles for protein and gene delivery. In experiments performed by Trapani *et al*. [66], phospholipid and chitosan containing nanoparticles were manufactured using the ionic gelation method in varying pH conditions for the pulmonary delivery of low molecular weight heparin. From *in vivo* studies in mice, they observed that nanoparticles manufactured in acidic conditions did not increase the coagulation time, while those prepared in a neutral condition had a significant increase in coagulation time in mice [66]. Multiple studies have been performed by using polymeric particles loaded with the anti-cancer compound, paclitaxel. In a recent study, paclitaxel-loaded polymeric micelles were produced using a combination of polyethylene glycol (PEG5000) and polymer poly(ethylene oxide)-block-distearoyl phosphatidylethanolamine (DSPE). These micelles were tested in *in vivo* models using intratracheal instillation, as well as intravenous administration routes. Polymeric paclitaxel micelles were also compared with commercially available taxol compound. It was found that the intratracheal instillation route had better drug absorption in comparison with the intravenous route of administration. Furthermore, target drug delivery was achieved by maximum drug localization in the lung tissue, as compared to other tissues. It was observed that polymeric paclitaxel had better drug release profiles as compared to taxol [35]. In another study, paclitaxel with an amphiphilic block copolymer was formulated using poly-glycolide-ɛ-caprolactone with PEG and tocopheryl succinate [36]. This block copolymer was incorporated with paclitaxel in order to improve the encapsulation efficacy along with an increased cellular uptake. The copolymer-paclitaxel was labeled with coumarin-6 in order to track the uptake of nanoparticles in an *in vitro* A549 cell model. Along with the uptake studies, cytotoxicity studies were performed by comparing a commercial taxol compound with this paclitaxel-block copolymer, which demonstrated higher cytotoxicity over taxol and the free paclitaxel compound. For polymeric nanoparticles, it is possible to improve the encapsulation efficacy of the drug along with an improved uptake by modification of the surface of the particles. Apart from using block copolymers, chitosan has also shown improvements in uptake and sustained drug release [82]. PEGylation of particles has also been proven to increase the uptake, encapsulation efficacy and sustained drug release [37]. PEGylation of the particles evades the macrophages and, hence, avoids being engulfed by phagocytosis [19]. Apart from the anti-cancer agents, many antioxidants have also been incorporated into polymeric nanoparticles [43]. In a study performed by Yoo *et al*., a novel anti-inflammatory compound, hydroxybenzyl alcohol (HBA) incorporated polyoxalate (HPOX), was formulated using PLGA-based polymeric nanoparticles. The HPOX nanoparticles were administered via the intratracheal route in ovalbumin-induced asthma mice models. They observed attenuation in the inflammatory response by a decrease in the levels of pro-inflammatory cytokines in the ovalbumin treated group [39]. These novel anti-inflammatory compound HPOX polymeric nanoparticles may have the potential for the treatment of airway inflammation and asthma.

Although polymeric particles may be biodegradable, their degradation rate must be analyzed. Additionally, toxicity profiles must be elaborately analyzed in various *in vitro*, *ex vivo* and *in vivo* models. Drugs that have been encapsulated in polymeric nanoparticles for pulmonary administration are illustrated in Table 2.

### 3.3. Liposomes

Liposomes are an attractive drug delivery system, especially for pulmonary applications, as it is prepared primarily from phospholipids, which are inherent in lungs. They are prepared using lung surfactants, phospholipids, cholesterol, *etc*. Liposomes possess sustained release properties, which enable the maximum drug effect over a prolonged time period. In the 1990s, the first liposomal product was introduced, which was purified bovine surfactant (Alveofact^®^) for acute respiratory distress syndrome (ARDS) in infants by pulmonary instillation. Later, amphotericin B-loaded liposomes were introduced (Ambisome^®^), yet not for pulmonary, but for parenteral application [43]. Inhaled liposomes are still a challenge. Maintaining the critical physical properties of liposomes after nebulization holds the key for a successful liposomal product [83]. Currently, two liposomal products in the last stage of clinical development are dry powder liposomes, Arikace^®^ (amikacin, Insmed, Monmouth Junction, NJ, USA) and Pulmaquin™ (ciprofloxacin, Aradigm Corp., Hayward, CA, USA), for the treatment of lung infections [41,42,54,84]. Arikace^®^, an amikacin liposomal preparation consisting of dipalmitoyl-phosphatidylcholine and cholesterol, is under a Phase 2 trial for the treatment of cystic fibrosis-associated lung infection of *Pseudomonas aeroginosa*. In a recent clinical study, a double-blind, randomized trial consisting of 105 patients with *P aeroginosa* infection was carried out [85]. The patients were divided into a once-daily group of amikacin aerosol and a placebo groups for 28 days. The authors observed a decreased density of *P. aeroginosa* in the sputum of the amikacin treated group *vs.* placebo. Furthermore, improvement in lung functions was observed in the amikacin treated groups against placebo. From the results, they found satisfactory safety and tolerability of amikacin, improved lung functions, along with the decreased density of *P. aeroginosa* in sputum of cystic fibrosis patients. Similarly, ciprofloxacin liposomes were tested for lung infections. In a study performed by Liu *et al*. [55], ciprofloxacin-loaded liposomes were prepared using phospholipids and cholesterol by the film method. These liposomes were found to have an average particle size of 350 nm and a high encapsulation efficacy of up to 93%. Furthermore, an *in vitro* drug release study was performed using simulated lung fluid (SLF) and saline solution as the release medium. The authors observed a higher cumulative release of ciprofloxacin-liposomes in SLF compared to saline solution. In *in vivo* experiments performed in rats, they found a higher drug targeting efficiency of ciprofloxacin-liposomes compared to ciprofloxacin solution [55]. Other liposomal formulations containing antioxidants are also formulated for acute oxidant-related lung injury [40,86]. Mustard gas and its derivatives, like 2-chloroethyl ethyl sulfide (CEES), are responsible for lung injury by disturbing the oxidant-antioxidant balance. In a study by Hoesel and colleagues, they produced phospholipid-based liposomes using antioxidants, like n-acetylcysteine, vitamin E and glutathione, by the film method. In an *in vivo* study with rats, a decrease in pro-inflammatory cytokine levels was observed in the bronchoalveolar lavage fluid (BAL) fluid of CEES-injured rats [86]. Such antioxidant containing nanoparticles can be delivered to the lungs either by nebulization or by instillation. Additionally, these antioxidant nanoparticles can certainly prove useful in the therapy for hypoxia and oxidative stress-related injuries of the lungs. Liposomes manufactured utilizing either physiological lipids or polymers as the base, in liquid form or as dry powder liposomes for reconstitution, have always been attractive candidates for the pulmonary delivery of various APIs. The APIs that have been incorporated as liposomal formulations are listed in Table 2.

## 4. Physicochemical Characterization for Nanoparticle-Based Systems

### 4.1. Particle Size and Zeta Potential Measurements

Particle size and zeta potential measurements are important for the characterization of nanoparticles in order to ensure the optimal particle size distribution and polydispersity index (PDI). The most commonly used particle size measurement techniques include photon correlation spectroscopy (PCS) and laser diffraction (LD). PCS normally measures particles ranging between a few nanometers to a maximum of 3 μm. The PCS technique measures the scattering of light by the dispersed particles, which move due to Brownian motion. PCS can also measure the PDI, which determines the uniformity of the particles. A higher value of PDI (above 0.2) normally indicates multiple sizes of particles in the given formulation. Hence, the smaller the PDI, the more uniformly the particles are distributed within the formulation. The LD technique measures particles with a bigger size and is based on the measurement of the diffraction angle depending on the particle radius. LD measures particles from the nanometer to a few millimeter size ranges [6,7]. It is recommended to use both techniques simultaneously. Zeta potential measurements are essential for predicting the stability of particles. The higher the zeta-potential, the higher is the repulsion between the particles, and as a result, there is less aggregation. The more uniformly the particles are distributed, the more stable is their shelf life [6].

### 4.2. Differential Scanning Calorimetry (DSC)

Differential scanning calorimetry (DSC) is one of the most important methods for the analysis of polymorphic changes in a lipid matrix. Structural changes in the lipid matrix provide information regarding the stability of the formulation over time. Melting and recrystallization curves are appropriate parameters to determine polymorphic changes in lipid matrices. A detailed analysis of the use of this technique in lipid-based systems is described by Bunjes *et al*. [87–89].

### 4.3. X-ray Diffraction

Along with DSC, X-ray diffraction is essential for analyzing the crystal structure and spacing in the lipid lattice. The incorporation of an API influences the lipid/polymer structure and spacing of the lattice. This method provides information of the patterns in the spacing and changes in the lipid/polymer structure and crystallinity can be mapped along with DSC. Hence, it is recommended to use both techniques simultaneously when analyzing any lipid-based formulations [6,89].

More advanced methods for physicochemical characterization include nuclear magnetic resonance (NMR), Raman spectroscopy and infrared spectroscopy. These techniques are more useful tools for the characterization of mixed systems, where different types of particles may co-exist (SLN, micelles, liposomes, liquid crystals) [6].

### 4.4. Microscopical Techniques/Particle Morphology

The morphology of the nanoparticles can be examined by using transmission electron microscopy utilizing different techniques suitable for specific particles. Freeze-fracture, negative staining and cryogenic-transmission methods can be adapted according to the type of particles. The interpretation of the morphology of the particle gives an idea about the structure, shape and alignment of the particles in the formulation. The interpretation of the size can also be confirmed using this method [88].

## 5. Cell- and Animal-Based Studies

Several studies have been performed for the toxicity analysis of the nanoparticle systems in different cell lines, tissue models, as well as animal models. Toxicity testing is essential in order to determine the lethal dose, as well as the therapeutic window of the drug-loaded nanoparticles. Apart from toxicity testing, therapeutic efficacy is also essential, and several models have been established that illustrate various disease conditions. Many *in vitro* models have been established using epithelial cells from the respiratory tract for testing nanoparticles after inhalation. Models involving an air liquid interface (ALI) have been extensively used to study the effects of various formulations intended for pulmonary application [90–95].

### 5.1. In Vitro Lung Epithelial Cell Culture Models

*In vitro* cell culture models are essential for the primary testing of any formulation before proceeding towards *ex vivo* and *in vivo* testing. *In vitro* cell models offer numerous advantages, such as continuous cell lines, easy handling and availability in large numbers, which offers the user many possibilities for designing multiple experiments simultaneously and, hence, limits the use of live animals. Different cell models derived from pulmonary epithelium from murine and human tissues have been established over the last two decades [92,94]. The main aspect is to develop a standard cell line that can predict the transporter mechanisms across the pulmonary epithelium in a similar fashion as the Caco-2 cell line, which has been standardized for transporter mechanism studies in the gastrointestinal tract. For this precise reason, many studies were performed using pulmonary epithelial cell lines derived from both human and murine sources as a lung-equivalent to Caco-2.

A human bronchial epithelium cell line (16HBE14o) has been used for a long time for studying the drug transport mechanism in airways. Forbes *et al*. established this cell line as a model for studying airway transport and drug permeation mechanisms. They studied different experimental parameters, like seeding density, transepithelial electric resistance (TEER), culture conditions necessary for optimal drug transport [91,92]. The barrier properties of the cell model were also studied using mannitol and transport and analyzing the TEER values [92]. They observed the higher permeability of hydrophilic molecules in the 16HBE14o cell line compared to the typical alveolar epithelium cell models. Furthermore, the permeability of lipophilic molecules was observed to have a sigmoidal relationship between permeability and lipophilicity.

Another pulmonary epithelium cell model was characterized by Foster *et al*. [95], Calu-3, a human sub-bronchial gland cell line. They studied the cell culture conditions, TEER values necessary to form tight monolayers necessary for drug permeation studies, as well as other parameters, like the drug transport and efflux of small molecules. Since then, Calu-3 has been extensively used to study drug permeation and transport mechanisms of small drug molecules and xenobiotics to be administered via the pulmonary route [95] The tight monolayer present in this cell line also expresses the cystic fibrosis transmembrane conductance regulator (CFTR) protein, which is essential for studies regarding cystic fibrosis. The presence of this protein has an added advantage in the testing of drugs for cystic fibrosis [95]. Furthermore, for Calu-3 cells, they can be cultivated in transwell plates with two compartments: a basolateral compartment with cell culture medium and an apical compartment, which is normally empty, representing air. Thus, an air-liquid interface (ALI) culture can be established. As in physiological conditions, pulmonary epithelium of this cultivation type is in contact with air and can be correlated with physiological conditions [95].

Other cell lines derived from human pulmonary epithelium (pneumonocytes Type-2) include the A549 cell line. Murine-derived cells include rat tracheobronchial cell lines and primary cells, rabbit primary alveolar-type cells and cell lines and many more [94].

### 5.2. Ex Vivo Lung Tissue Models

*Ex vivo* lung tissue models have been used extensively along with the *in vitro* and *in vivo* models. *Ex vivo* models can be advantageous for studying drug transport mechanisms across lung tissue and can provide information with respect to *in vitro-in vivo* correlation. Different *ex vivo* models were established, such as isolated perfused lung (IPL) and precision cut lung slices (PCLS).

IPL are prepared from mostly murine models, *i.e.*, rats. Mice are seldom used, owing to their small size and the difficulty in the isolation procedure. Other animals commonly used include rabbits and guinea pigs. The IPL model includes a complete lung that has been isolated from the body and immersed in an artificial system that resembles physiological conditions. IPL is encased in a system containing physiological buffer solutions (Krebs–Ringer or Krebs–Henseleit), where a 37 °C temperature condition is maintained. The perfusate flow is maintained between 12–15 mL/min. The perfused solution is also equilibrated with a mixture of oxygen and carbon dioxide to ensure the proper functioning of the lung tissue. The advantage of the IPL model is that it eliminates the first pass effect influence and retains most of the physiological properties of the lung tissue; hence, it is much closer to the *in vivo* system in comparison to the *in vitro* lung cell model. However, IPL requires great skill and precision for removing the intact lungs from the animal. Other experimental challenges, like mounting the tissue and maintaining an experimental physiological condition, are of paramount importance for success in experimentation. The IPL model with a detailed experimental set up and challenges is described by Sakagami [94]. Beck-Broichsitter *et al*. used this model for studying the uptake of nebulized 5(6)-carboxyfluorescein (CF)-loaded polymeric nanoparticles in an IPL *ex vivo* model. In this study, the absorption of CF nanoparticles in the perfusate solution in the IPL model was analyzed for the amount of CF transported into the lungs [4]. In this study, the authors compared the absorption and uptake of CF in nanoparticles *vs.* CF in solution in the rabbit IPL model. They observed a higher concentration of CF in the perfusate of the CF solution compared to CF nanoparticles. They also analyzed the physicochemical aspects of CF-loaded nanoparticles, such as the particle size and nebulization performance. It was found that the nebulization of CF nanoparticles had no influence on the particle size and the polydispersity index.

Another most convenient *ex vivo* model is precision cut lung slices (PCLS). PCLS can be prepared using murine models (rats and mice). For PCLS, the lungs are filled with a low-melting agarose solution. The agarose filled lungs are maintained in cold conditions to allow gelation, and the lungs are then sliced to the desired thickness using a tissue slicer. The entire slicing process is performed in cold cell culture medium, ensuring that the viability of the slices is maintained. The slices are then washed a minimum of three to four times using cell culture medium to remove traces of agarose. The slices can also be cut in specific positions, e.g., slices, including alveoli and pulmonary vessels. This way, the slices can be utilized for experiments with drugs involving the contraction and relaxation of the pulmonary vessels. In this way, the contraction-relaxation intensity can be observed by video microscopy, and special software calculations are performed using the area of contraction-relaxation of the vessel. The slices being the actual lung tissue retain most of the physiological properties and receptor mechanisms, along with the inflammatory responses and are viable up to three days. Toxicity testing of sildenafil-loaded SLN and plain SLN was performed using PCLS as an *ex vivo* model [9,10,96,97].

### 5.3. In Vivo Models

*In vivo* models involve whole animals for drug absorption and disposition studies in the lungs. The commonly used animals in *in vivo* experiments include small rodents, like mice, rats and guinea pigs. Mice being much smaller than the others are challenging for the removal of blood samples and lung dosimetry. Other larger and much more expensive animals include pigs, rabbits, sheep and monkeys, where studies with regard to inhalation pharmacokinetics, formulation and device efficiency are concerned. The most commonly used method is intratracheal instillation. In intratracheal instillation, the animal’s trachea is exposed, and through an endotracheal tube, an incision is made between the tracheal rings. Through this bifurcation, a typical volume between 10–200 μL of an aqueous suspension or solution of the test formulation is instilled using a microsyringe [94]. Apart from intratracheal instillation, animal can be directly exposed to the aerosol in a chamber where the nose of the animal is fixed, and then, the animal inhales the released aerosol (the nose only model). After exposure for a specified time, the animal is sacrificed, and the bronchoalveolar lavage fluid (BAL) is analyzed for the desired components. Studies involving SLN aerosol were performed by Nassimi *et al*. In their studies, they exposed Balb/c mice to SLN aerosol for a period of 16 days, and cytotoxicity studies were performed by investigating BAL fluid and cytokines, along with histopathological evaluation of the lungs [8,9]. Intratracheal instillation involves skill in tracheotomy surgery. Furthermore, multiple blood samples from the animal are essential in order to determine the kinetics of the drug formulation. In comparison, the nose only model is relatively easier for the animal, as well as it enables the investigator to have flexibility. Euthanization of the animal, followed by the differential count in BAL fluid is a lot faster than multiple blood sample analysis. Apart from the procedure itself, many experimental parameters should be considered, like the animal, breathing frequency, device specifications, flow rate, inter-animal variations and uptake mechanisms.

Many *in vivo* models have been established with respect to different lung disease conditions. The monocrotaline sodium-induced pulmonary hypertension model, bacterial infection models for anti-tuberculosis formulations, the lipopolysaccharide (LPS)-induced airway inflammation model, acrolein-induced airway inflammation and mucus production in murine models are some of the models developed for *in vivo* testing [98–100].

*In vivo* animal experiments are particularly important in preclinical studies and help to determine the safe dosage range of a formulation for the subsequent Phase 1 of the clinical trial on humans.

## 6. Toxicity Assays

*In vitro* and *ex vivo* toxicity testing involves many assays for assessing the toxicity of drug formulation. Different cell targets can be utilized, such as mitochondria, cell membrane integrity, nuclei staining, lysosomal activity and DNA ladder assays for apoptosis cell death mechanisms, *etc*. The 4,5-dimethylthiazol-2-yl)-2,5-diphenyltetrazolium bromide (MTT) assay has been established for more than two decades as the standard for cell viability measurements in a variety of cell lines. Based on the similar principle of the metabolic activity of mitochondria, other assays involving tetrazolium salts, resazurin and the neutral red test can also be used to assess cell viability. Comprehensive data of the most commonly used assays for *in vitro* and *ex vivo* studies have been described by Arora *et al*. [101]. For studies involving a specific disease condition of the lungs, specific assays should be selected in order to achieve relevant data. Cytokine activation, e.g., TNF-α, interleukins and prostaglandin measurements, should also be performed to analyze the safety of a formulation. Polymeric nanoparticles require special attention, being synthesized from partially synthetic materials; they may have the potential for the activation of cytokines. Solid lipid nanoparticles, although prepared with physiological phospholipids, still should be thoroughly tested for toxicity and inflammatory cytokine response in different cell lines and lung tissues, because contamination with bacterial endotoxins has to be excluded.

Toxicity assays are a key guideline for the preclinical phase and provide vast information regarding the safety of the drug formulation, as well as the possible safe dose for *in vivo* animal studies. Data obtained from the *in vitro*, *ex vivo* and *in vivo* testing should be scrutinized thoroughly along with multiple experimental parameters. Multiple assays for *in vitro* and *ex vivo* experiments are recommended to avoid errors and to improve statistical analysis. For example, the MTT and resazurin assays are based on a similar principle of mitochondrial activity and, hence, should be run in parallel in order to reduce errors and improve data analysis. Assays with different mechanisms can also provide more information and should be performed simultaneously [93].

## 7. Conclusions

Pulmonary drug delivery is rapidly gaining importance, due to the multiple advantages. Lungs offer a vast variety of advantages over conventional oral drug delivery. The large surface area and the elimination of the first past effect makes the absorption of drugs in the lungs faster. The size of these colloidal systems being in the nanometer range certainly gives them an advantage over conventional dosage forms. Poorly soluble drugs can be incorporated in various colloidal systems. Lipid-based colloidal systems namely, SLN and liposomes, have an added advantage owing to their physiological components in the formulation. Biodegradable polymeric nanoparticles may exhibit a sustained release effect, although the drug release mechanism still remains unclear and under scrutiny. Degradation rates of the polymers should also undergo a thorough investigation in order to substantiate such claims. Although many studies have been established for testing the safety of nanoparticle-based colloidal systems, they still largely remain in the preclinical phases of drug development, and only a selected few colloidal systems are available in the market. In spite of the skepticism, there are advances with respect to the formulation of liposomes using the freeze drying process and the production of dry powder liposomes for reconstitution. These dried liposomal formulations are paving their way towards Phase 3 clinical studies, and hopefully, successful commercialization of nanoparticles for inhalation can be achieved. Additionally, the technology and development of an appropriate nebulization device is equally essential. The field of aerosol devices should also focus in parallel on the development of an appropriate device according to the particle type and API incorporated. Apart from the physicochemical characterization of nanoparticle-based systems, parameters, like the device type, the flow rate, the volume administered and other technical constraints, also have an influence on the success and commercialization of the formulation. Cell-based studies are equally important to find a safe dosage range and the toxic concentrations in various cell and tissue models. The most challenging part is the *in vitro*/*ex vivo* to *in vivo* correlation. The interpretation of the results should be carried out with precision, so that *in vitro* and *in vivo* can be comparable. Additionally, with the formulation characterization, the most important aspects are the uptake and clearance mechanisms of a formulation in the lung cell. There are still blanks for the exact mechanism of transport of drugs across the pulmonary epithelium that needs to be filled. Radioisotope labeling and fluorescent labeling may allow for tracing the uptake of the drug into the cell, but the correlation with realistic disease state models is still unknown. To conclude, pulmonary research has vast opportunities for the success of nanoparticulate systems, which needs thorough physicochemical and nanotoxicological analysis for possible human application.

## Figures and Tables

**Table 1.** Correlation between the areas of lung deposition, the mechanism of deposition and particle size [17].

Location	Size	Mechanism
Primary Bronchi	5–10 μm	Impaction
Secondary Bronchi	1–5 μm	Sedimentation
Bronchioles	1–3 μm	Sedimentation
Alveoli	0.5–1 μm	Brownian motion

**Table 2.** Different active pharmaceutical ingredient (API) molecules incorporated into different nanoparticle systems for pulmonary application. SLN, solid lipid nanoparticles; SLM, solid lipid microparticles; NP, nanoparticles; NC, nanocarriers.

Disease/API category	Type of particles	Reference
**Anti-asthma/anti-inflammatory**		

Beclomethasone	Lipid NC	[25]
Budesonide	SLN, liposomes	[26,27]
Curcumin	SLN, polymeric NP	[22,28]
Indomethacin	Lipid NP	[29]
Fluticasone	Dried NP	[30]
Pirfenidone	Polymeric NP	[31]

**Anti-cancer**		

Cisplatin	Dried NP	[32,33]
Methotrexate	Polymeric NP	[34]
Paclitaxel	Polymeric NP	[35–37]
Silibinin	SLN	[38]

**Anti-oxidants**		

Antioxidants-multiple types	Liposomes, polymeric NP, SLM	[21,39,40]

**Lung infections**		

Amikacin	Liposomes, SLN	[23,41,42]
Amphotericin B (Ambisome^®^)	Liposomes (parenteral)	[43]
Anti-tuberculosis drugs	SLN, polymeric NP, Liposomes	[27,44–53]
Ciprofloxacin	Liposomes	[54,55]
Moxifloxacin-Ofloxacin	Dried NP, MP	[56]
Tobramycin-Clarithromycin-Vancomycin	Spray dried NP, MP	[57–59]
Voriconazole	Polymeric NP	[60]
Tacrolimus	Lipid NP	[61]
Itraconazole	Lipid NC, dried NP	[62,63]

**Proteins, peptides and macromolecules**		

Calcitonin	Polymeric liposomes	[64,65]
Heparin	Polymeric NP	[66]
Insulin	SLN	[67,68]
Exendin-4	Polymeric NP	[69]

**Pulmonary arterial hypertension/Congestive heart failure**		

Iloprost	Liposomes	[70]
Sildenafil	Polymeric NP, SLN	[10,71]
Carvedilol	Polymeric NP	[72]

**Surfactant, gene and antibody delivery**		

siRNA/gene	Polymeric NP	[73,74]
Surfactant therapy	Liposomes	[75–79]
DNA vaccine	Polymeric liposomes	[80]
IgG1	Self-assembly NP	[81]
